# Evaluation of physical and mental health conditions related to employees’ absenteeism

**DOI:** 10.3389/fpubh.2023.1326334

**Published:** 2024-01-11

**Authors:** Kazumitsu Nawata

**Affiliations:** Hitotsubashi Institute for Advanced Study (HIAS), Hitotsubashi University, Kunitachi, Japan

**Keywords:** absenteeism, absence days, physical and mental health, medical checkups, job stress

## Abstract

**Background:**

Employees’ health conditions are issues for not only employees themselves but also companies and society to keep medical costs low and productivity high.

**Data and methods:**

In this analysis, 15,574 observations from 2,319 employees at four operational sites of a large corporation were used. The dataset contained physical and mental health conditions obtained from annual mandatory medical checkups, the Brief Job Stress Questionnaire (BJSQ), and work record information. Health and other factors related to long-term absenteeism (over three days in a quarter) were analyzed. Data were collected between February 2021 and January 2022, and we converted into quarterly observations. A logit (logistic regression) model was used in the analysis.

**Results:**

Age and gender were identified as important basic characteristics. The estimates for these variables were positive and negative and significant at the 1% level. Among the variables obtained from the medical checkups, the estimates for diastolic blood pressure, HbA1c, anamnesis, heart disease history, smoking, increased weight, and frequency of alcohol consumption were positive and significant at the 1% level, further those for taking antihypertensive medications and kidney disease history were positive and significant at the 5% level. In contrast, the estimates for systolic blood pressure and amount of alcohol consumption were negative and significant at the 1% level. The estimate for taking antihyperglycemic medications and health guidelines were negative and significant at the 5% level. Among the variables obtained from the BJSQ, the estimates for amount of work felt, fatigue and support from family and friends were positive and significant at the 1%, and the estimate for irritation was positive and significant at the 5% level. The estimates for controlling job and physical complaints were negative and significant at the 1% level, and those for usage of employee’s ability to work and suitability of the work were negative and significant at the 5% level. As all four operational sites were located in the northeastern region of Japan (cold and snowy in winter), the seasonal effects were significant at the 1% level. The effect of year was also significant and significant differences were observed among the sites at the 1% level.

**Conclusion:**

Some physical and mental health conditions were strongly associated with long-term absenteeism. By improving these conditions, corporations could reduce the number of employee absence days. As absenteeism was costly for corporations due to replacement employees and their training costs to maintain operations, employers must be concerned about rising healthcare (direct and indirect) costs and implement investments to improve employees’ health conditions.

**Limitations:**

This study’s results were based on only one corporation and the dataset was observatory. The employees were primarily operators working inside the building and most of them are healthy. Therefore, the sample selection biases might exist, and the results cannot be generalized to other types of jobs, working conditions, or companies. As medical checkups and the BJSQ are mandatory for most companies in Japan, the framework of this study can be applied to other companies. Although we used the BJSQ results, better mental measures might exist. Similar analyses for different corporations are necessary.

## 1 Introduction

The International Labour Organization (ILO) (1) estimated that losses due to health problems would account for approximately 3.94% of annual global GDP. The World Health Organization (WHO) (2) reported that the economic loss caused by work-related health problems [any illness caused or made worse by workplace factors (3)] would be 4%–6% of GDP in most countries. Maintaining and improving employee health are serious issues for employers. WHO (2) also mentioned that “workplace health initiatives can help reduce sick leave absenteeism by 27% and health-care costs for companies by 26%”. Several studies have been conducted on the productivity, characteristics, and health conditions of employees (4–17). Various authors have also evaluated monetary costs and returns on health investments (18–21). Loeppke et al. (22) stated that health-related productivity costs were over four times higher than medical costs. In their analysis, they developed a database by integrating the medical and pharmacy claims data with the productivity and health information obtained from the 15,380 Health and Performance Questionnaire (HPQ) respondents of four companies. Then, they added information collected on employer business measures to the database.

Health-related productivity losses have been attributed to absenteeism (repeatedly being absent from work due to health problems) (23) and presenteeism (being present at work but with reduced productivity due to health conditions) (24). Presenteeism is a complicated problem (25) and its proper measurement is difficult. Worker absence is a good proxy for employees’ health conditions (26). Since most of the corporation’s employees clock in and out of work, and additional trained employees are required to maintain corporate operations, absenteeism is the cornerstone metric guiding corporate policy for healthcare investment (27). Nawata (28) evaluated the health factors affecting absenteeism using data obtained from 1,136 employees at one operational site of a large corporation. However, this study has the limitations: (i) the number of observations was not large and the observation period was just three months, and (ii) only limited factors of physical health conditions obtained from medical checkups were used, ignoring the factors representing mental health conditions. Mental health is important for employee well-being, productivity, and absenteeism (29–42). Goetzel et al. (43) emphasized that employers must be concerned about rising mental healthcare costs. Bryan et al. (44, p.1519) found “that a change in mental health has an effect on absenteeism more than three times greater than a change in physical health”.

Since 2015, annual stress checks have become mandatory for companies with 50 or more workers in Japan under the Amendments of Industrial Safety and Health Act (45). The Japanese government also launched the Stress Check Program to screen workers with high psychological stress in the workplace (46). These amendments aim to prevent workers’ mental disorders and improve working conditions that might cause job stress. Medical checkups and stress checks are performed as part of the regular operations of companies, and all costs are paid by the companies. That is, not only all direct costs but also necessary times for medical checkups and stress checks are treated as paid working hours. Hence, employers must be aware of these results to improve employee health. Tsutsumi and Kawakami (46) mentioned that the Japanese Stress Check Program might be effective in improving workers’ mental health. The Brief Job Stress Questionnaire (BJSQ) (47) is usually used for stress checks, in which each worker answers 57 job stress questions. Watanabe et al. (48) also reported that the BJSQ helped to measure psychosocial factors at work. Therefore, we used the BJSQ results to represent the employees’ mental health conditions. The BJSQ comprises four parts: job concerns, health conditions, people around the worker, and satisfaction. The 57 questions are then summarized into 19 items scored from 1 to 5; a higher score represents better conditions, that is, 5 is the best and 1 is the worst (49).

In this study, both physical and mental health conditions related to absenteeism were analyzed using 15,574 observations obtained from 2,319 employees over the period of February 2021 to December 2022. To the best of the author’s knowledge, this study is the first attempt analyzing the relationship using a large individual dataset.

## 2 Data and models

### 2.1 Data

The dataset contained information on medical checkups, BJSQ answers, and work records obtained from employees at four operational sites of a large corporation. Most employees were operators helping end consumers of client companies through telephones or the Internet at indoor operational sites. The sample period was from February 2021 to December 2022. Work records included information on work schedules, actual work hours, and employee absences. As seasonal factors (especially cold and snow in winter) matter in the locations of the sites, the sample period was divided into eight quarters: the first quarter (Q1) of 2021 to the fourth quarter (Q4) of 2022, and quarterly absence days were considered. Absence days were obtained from non-working days due to disability (sick or injured) and personal reasons. Paid, maternity and parental, nursing care, bereavement, auspicious, and special leaves absence days admitted by the corporation’s regulations were excluded.

**Figure 1:**
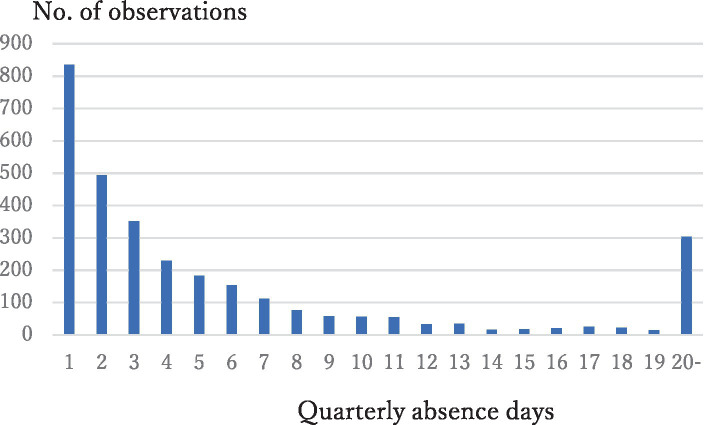
Distribution of quarterly absence days.

The distribution of absence days has a heavy right tail (Figure 1). We defined long-term absenteeism as the absence of an employee for over three days in a quarter (more than one day per month), following Nawata (28). As Q1 of 2021 only contained two months of working records, and the reasons for absences were not available for one month in Q4 of 2021 at one site, we defined long-term absenteeism if an employee was absent for over two days in those cases. Some employees resigned and some were hired during the sample period. As we consider quarterly data, these errors would be smaller than the annual data case. Working records were combined with annual medical check-ups in the same year. If an employee underwent two or more medical check-ups per year, the latter result was used. A total of 2,765 employees underwent medical checkups at least once during the study period. Among them, 2,699 employees had absence day data for at least one quarter of the year they underwent medical checkups, and we obtained 18,549 (person-quarter) observations. Figure 1 shows the distribution of quarterly absence days. The total number of absence days was 24, 619. A total of 15,456 or 83.3% of observations had no absence days. However, 1,459 or 7.9% of observations classified as long-term absenteeism accounted for 21,880 or 88.9% of total absence days. Hence, reducing long-term absenteeism is important for employers. A total of 16,660 observations from 2,409 employees provided BJSQ answers in the same year. After excluding observations with missing data, 15,574 observations obtained from 2,319 employees were used in the final model.

### 2.2 Models

We set three indices determined by employee, year, and quarter given by $\left(i,t,q\right)$ and converted the data into quarterly (one dimensional) observations. We define $L:\mathrm{Absenc}e_{itq}$ =1 if the *i*-th employee has long-term absenteeism at the *q*-th quarter of year t and 0 otherwise. The continuous integer index (hereafter, observation number) $\ell =g\left(i,t,q\right)$ , ℓ=1,2,…,n , was assigned for each $\left(i,t,q\right)$ . Note that the assignment is one to one and $\left(i,t,q\right)$ is uniquely determined when ℓ is given. Let $q_{1}$ the final quarter that the (*i*-1)-th employee worked and $q_{2}$ be the first quarter that *i*-th employees worked at year t, $\ell_{1}=g\left(i-1,t,q_{1}\right)$ and $\ell_{2}=g\left(i,t,q_{2}\right)$ . If these employees worked throughout the year, $q_{1}=4$ and $q_{2}$ = 1. The observation number is assigned so that $\ell_{1}+1=\ell_{2}$ and $\ell_{2}+1=g\left(i,t,q+1\right)$ if the *i*-th employee had worked in both *q* and *q* + 1 quarters in year t. Let $n_{1}$ be the number of observations in year *t*. Then, the observation number starts from $n_{1}+1$ in the next year. We obtained 18,549 (person-quarter) observations.

Of these observations, 7.9% were $L:\mathrm{Absenc}e_{\ell }$ =1. The basic model used in the analysis is the logistic regression (logit) model with the fixed time effect given by


(1) $$P\left[L:\mathrm{Absenc}e_{\ell }=1\right]=\Lambda \left(x_{\ell }^{\prime }\beta +\gamma_{tq}\right),\;\ell =1,\;2,\ldots ,\;n\text{,}$$

where Λ is the distribution function of the logistic distribution given by Λ(ω) = (exp(ω))/(1 + exp.(ω)); $x_{\ell }$ is a vector of covariates representing the employee’s characteristic and health condition; *β* is a vector of unknown parameters; $\gamma_{tq}$ is the fixed time effect, and *n* is the number of all observations. Since the medical checkup and BJSQ results are available only once a year, we assume that $x_{\ell }$ does not change in year *t* so that $x_{\ell }\text{≡}x_{tiq}=x_{ti}$ for any possible 1≤q≤4 where $x_{ti}$ represents the medical checkup and BJSQ results of the *i*-th employee in year *t*. Table 1 shows the assignment example of the observation number ℓ and $x_{\ell }$ when employees worked thought the year. Here after, we omit the subscript ℓ to avoid unnecessary complications.

**Table 1:** Assignment example of observation number ℓ in year t and values of $x_{\ell }$ when employees worked throughout year *t*.

Employee number	*i*-1	*i*				*i* + 1
Quarter *q*	… 4	1	2	3	4	1 …
Observation number ℓ	… $\ell_{0}$	$\ell_{0}+1$	$\ell_{0}+2$	$\ell_{0}+3$	$\ell_{1}=\ell_{0}+4$	$\ell_{1}+1\ldots$
$x_{\ell }$	… $x_{i-1,t}$	$x_{\mathrm{it}}$	$x_{\mathrm{it}}$	$x_{\mathrm{it}}$	$x_{\mathrm{it}}$	$x_{i+1,t}\ldots$

### 2.3 Selection of covariates

As shown by Nawata (50), the selection of covariates is important. If we do not add the appropriate covariates, we obtain misleading results. However, if we add covariates that are irrelevant to absenteeism, we may lose the efficiency of the estimation due to multicollinearity among covariates and a reduction in the number of observations by missing values. The number of factors obtained from the medical checkups and the BJSQ was 41 and 19, respectively. Quarter, site location and year dummies were the other potential covariates. Therefore, it was necessary to control for the number of covariates.

The basic characteristics of employees are as follows:

*Female* (dummy variable) is 1 if female and 0 if male, and *Age* (age of an employee).

Since these factors were fundamental, not affected by the health conditions and highly significant in all models, we selected health factors based on the models with these factors.

We employed the procedure used in Nawata (28) to select the proper medical checkup and BJSQ covariates. The dataset was observatory and causality problems might exist, and we analyzed the variables possibly related to absenteeism. The procedure is based on likelihood ratio statistics and the Akaike information criterion (AIC), one of the most widely used criteria in model selection. It is important to use likelihood ratio statistics because t-test statistics may provide misleading results in binary choices and similar models (51, 52). Let $x_{1},x_{2},\ldots ,x_{k}$ be (potential) covariates. The medical checkup covariates were selected by the following stepwise procedure that increases the covariates one by one:

- Estimate the model given by

(2) $$P\left[L:\mathrm{Absence}=1\right]=\Lambda \left(\beta_{0}+\beta_{1}\mathrm{Age}+\beta_{2}\mathrm{Female}\right)\text{,}$$

$$\begin{array}{l} P\left[L:\mathrm{Absence}=1\right]=\Lambda \left(\beta_{0}+\beta_{1}\mathrm{Age}+\beta_{2}\mathrm{Female}+\beta_{3}x_{j}\right),\\ j=1,2,\ldots ,k\text{.} \end{array}$$

Let the log likelihoods of the first and second equations and their difference be $\log L_{0}$ , $\log L_{1j}$ and $LR_{1j}=\log L_{1j}-\log L_{0}$ for i=1,2,…,k in Eq. (2) using without missing values. $2\cdot LR_{1j}$ is the likelihood test statistic of $H_{0}:\beta_{3}=0$ and asymptotically follows $\chi^{2}\left(1\right)$ under the null hypothesis. Choose $x_{j}$ that maximizes $LR_{1j}$ .

- Without a loss of generality, we can assume that the first variable $x_{1}$ maximizes $LR_{1j}$ .

Let


(3) $$P\left[L:\mathrm{Absence}=1\right]=\Lambda \left(\beta_{0}+\beta_{1}\mathrm{Female}+\beta_{2}\mathrm{Age}+\beta_{3}x_{1}\right)\text{,}$$

$P\left[L:\mathrm{Absence}=1\right]=\Lambda \left(\beta_{0}+\beta_{1}\mathrm{Female}+\beta_{2}\mathrm{Age}+\beta_{3}x_{1}+\beta_{4}x_{j}\right)\text{,}$ j=2,3,…,k, and calculate the second stage log likelihoods an the difference, $\log L_{1j}$ , $\log L_{2j}$ and $LR_{2j}=\log L_{2j}-\log L_{1j}$ for j = 2,3,…, k in Eq. (3) using observations without missing values. Let $x_{2}$ be a variable that maximizes $LR_{2j}$ .

iii) Repeat steps m +1 times by increasing covariates one by one until $LR_{m+1j}<1$ for all j>m It corresponds to minimizing the AIC. The selected model becomes Eq. (4) given by


(4) $$P\left[L:\mathrm{Absence}=1\right]=\Lambda \left(\begin{array}{l} \beta_{0}+\beta_{1}\mathrm{Female}+\beta_{2}\mathrm{Age}+\beta_{3}x_{1}\\ +\beta_{4}x_{2}+\cdots +\beta_{m+2}x_{m} \end{array}\right)\text{.}$$

This procedure allowed to select the following variables from the medical checkups. The deals of the section procedure are available upon request to the author.

*SBP* (systolic blood pressure) mmHg,

*DBP* (diastolic blood pressure) mmHg,

*GOT* (glutamic-oxaloacetic transaminase) units per liter (U/L),

*GPT* (glutamic-pyruvic transaminase) U/L,

*Triglyceride* (serum triglyceride level) mg/dL,

*HDL* (high-density lipoprotein cholesterol) mg/dL,

*Hb*A1c (hemoglobin A1c) %,

*Anamnesis* (dummy variable) is 1 if having anamnesis of any disease and 0 otherwise,

*M_BP* (dummy variable) is 1 if taking antihypertensive medications to control blood pressure and 0 otherwise,

*M_Glucose* (dummy variable) is 1 if taking antihyperglycemic medications to control glucose and 0 otherwise,

*CBD* (dummy variable) is 1 if there is a history of cerebrovascular disease and 0 otherwise,

*Heart_D* (dummy variable) is 1 if there is a history of heart disease and 0 otherwise,

*Kidney_D* (dummy variable) is 1 if there is a history of kidney disease and 0 otherwise,

*Anemia* (dummy variable) is 1 if having anemia and 0 otherwise,

*Smoke* (dummy variable) is 1 if smoking and 0 otherwise,

*Weight_20* (dummy variable) is 1 if weight increased by 10 kg or more from age 20, and 0 otherwise,

*Exercise* (dummy variable) is 1 if exercising for 30 min or more twice or more in a week for more than a year, and 0 otherwise,

*Chew_Food* (can chew food items; integer 0–2) is 0 if everything, 1 if something, and 2 if difficult to chew,

*Eat_Fast* (eating speed; integer 0–2) is 0 if eating slower than other people, 1 if eating normally, and 2 if eating faster than others,

*Alcohol_Freq* (frequency of alcohol intake; integer 0–2) is 0 if never, 1 if sometimes, and 2 if every day,

*Alcohol_Amount* (amount of alcohol intake; integer 0–4) is 0 if none, 1 if drinking less than 180 mL of Japanese sake wine (with an alcohol percentage of approximately 15%) or equivalent alcohol per day when drinking, 2 if drinking 180–360 mL, 3 if drinking 360–540 mL, and 4 if drinking 540 mL or more),

*Sleep* (dummy variable) is 1 if sleeping well and 0 otherwise,

*H_Guidance* (dummy variable) is 1 if will take health guidances and 0 otherwise.

The following variables were selected from the BJSQ (stress check) answers using the same procedure of the medical checkup case. These variables take integers 1–5; a larger value is better (stress is less), 1 is the worst, and 5 is the best. Tsutsumi et al. (53) considered the cut-off points to identify the high-stress employees. Since the cut-off points were obtained from the BJSQ answers and we assumed that they associated with absenteeism continuously, we directly used the values of these items in the analysis.

*M_Burden* (mental burden concerning to quantity of work),

*S_P_Burden* (subjective physical burden),

*Control_Work* (control level of work),

*Ability_Usage* (utilization of knowledge and skills at work),

*W_Suitability* (suitability of the work for an employee),

*Reward* (rewarding work),

*Irritation* (irritation),

*Fatigue* (fatigue),

*Depression* (depression),

*P_Complaint* (physical complaints),

*C_Support* (support from co-workers),

*F_Support* (support from family and friends), and.

*Satisfaction* (work and family life satisfaction).

Since the year and seasonal factors were important, we considered that the time effect consisted of year and quarter effects and given by $\gamma_{tq}=\zeta_{t}+\eta_{q}$ . The following dummy variables were used to represent the effects of the year, season, and site:

*Y22* (year dummy) is 1 if year 2022 and 0 if 2021,

*Q1, Q3,* and *Q4* (quarter dummies representing the first, third, and fourth quarters, respectively. The base is the second quarter, where the probability of long-term absence is the lowest), and.

*Site2, Site3, Site4* (site dummies representing the second, third, and fourth sites. The base is the first site with the largest number of employees is largest).

Forty-five covariates were used in the analysis and *x’β*+*γ*_*tp*_ in Eq. (1) becomes Eq. (5) given by


(5) $$\begin{array}{c} x^{\prime }\beta =\beta_{0}+\beta_{1}\mathrm{Female}+\cdots +\beta_{25}\;M_{-}\mathrm{Burden}+\cdots \\ \;+\beta_{38}Y22+\cdots +\beta_{45}\mathrm{Site}4. \end{array}$$

**Figure 2:**
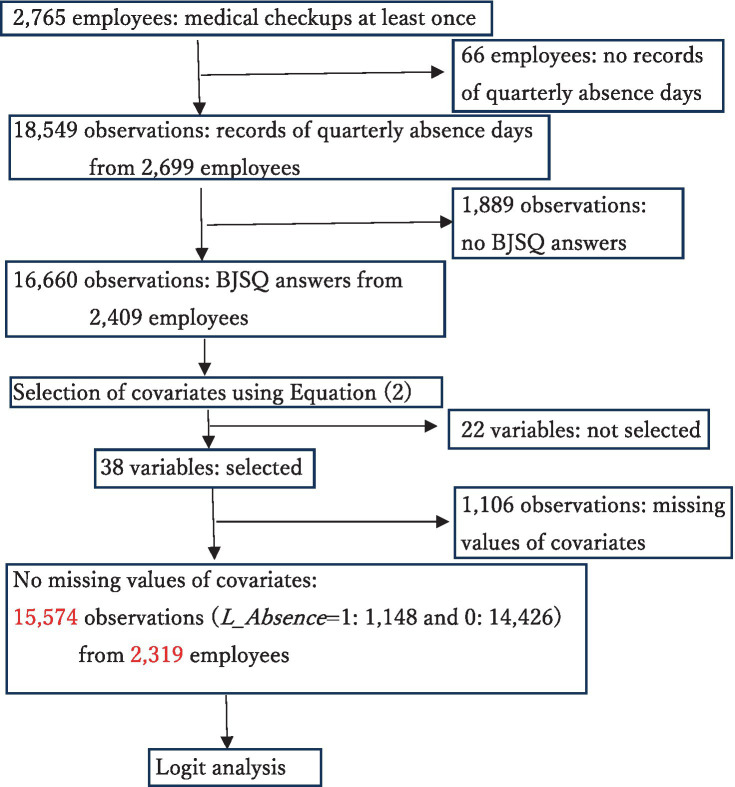
Flow chart of the study.

The study design is summarized in Figure 2, and the variables not used in the analysis are listed in Appendix A. A summary of the covariates is provided in Table 2. The list of abbreviations used in the study is given in Table A1 in Appendix B. The total number of observations used in the estimation of Eq. (3) is 15,574, of which 1,148 have *L_Absence* = 1 and 14,426 have *L_Absence* = 0.

**Table 2:** Summary of covariates.

Variables obtained from medical checkups			Variables obtained from BJSQ and quarter, site, year dummies		
Variable	Mean	SD	Variable	Mean	SD
*Female*	0.74		*M_Burden*	2.77	0.91
*Age*	39.34	10.51	*S_P_Burden*	3.51	0.71
*SBP*	124.32	19.70	*Control_Work*	2.89	0.93
*DBP*	74.63	13.41	*Ability_Usage*	2.69	0.75
*GOT*	23.28	13.90	*W_Suitability*	2.66	1.00
*GPT*	26.30	28.53	*Reward*	2.69	1.03
*Triglyceride*	101.47	78.47	*Irritation*	3.09	1.08
*HDL*	60.53	14.70	*Fatigue*	2.71	1.02
*HbA1c*	5.48	0.62	*Depression*	2.96	1.16
*Anamnesis*	0.569	0.50	*P_Compaint*	2.84	1.11
*M_BP*	0.085		*C_Support*	2.75	0.98
*M_Glucose*	0.031		*F_Support:*	3.14	1.36
*CBD*	0.007		*Satisfaction*	2.88	0.84
*Heart_D*	0.021		*Y22*	49.3%	
*Kidney_D*	0.002		*percent of observations by quarter*	Q1:24.1%, Q2:25.1%, Q3:25.4%, Q4:25.4%	
*Anemia*	0.250		*percent of observations by site*	Site1:51.6%, Site2:6.9%, Site3: 30.3%, Site4: 11.2%	
*Smoke*	0.241				
*Weight_20*	0.382				
*Exercise*	0.114				
*Chew_Food*	0:84.65%, 1:15.27%,2:0.08%				
*Eat_fast*	0:12.8%, 1:58.6%, 1:28.5%				
*Alcohol_Freq*	0:17.1%, 1:60.4%, 2:22.5%				
*Alcohol_Amount*	0:45.6%, 1:26.2%, 2:19.9%, 3:6.3%m 4:2.0%				
*Sleep*	0.569				
*H_Guidance*	0.253				

SD: standard deviation.

## 3 Results of estimation

**Figure 3:**
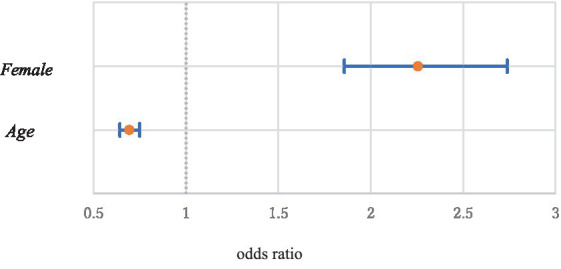
Odds ratios and 95% confidence intervals of *Female* and *Age* (Age 30 vs. Age 40).

Table 3 presents the estimation results. In the analysis, we used EViews 12. The gross percentage of long-term absenteeism (*L_Absence* = 1) was 7.4%, McFadden’s *R*^2^ was 0.0921, and the likelihood ratio statistic of the equation was 775.05. Among the basic characteristics, the estimate for *Female* was positive, and its t-value was quite large and highly significant. The odds ratio (OR) for females compared to males was 2.26 with a 95% confidence interval (CI) ranging from 1.86 to 2.74. The estimate for *Age* was negative and significant at the 1% level. The OR comparing employees aged 30–40 years was 0.69, with a 95% CI of 0.69–0.75. Figure 3 shows the ORs and 95% CIs for *Female* and *Age*.

**Table 3:** Results of estimation.

Variable	Coefficient	SE	Variable	Coeffcient	SE
Constant	−2.3945	0.5017	*M_Burden*	0.3398	0.0402^**^
*Female*	0.8132	0.0991^**^	*S_P_Burden*	0.0386	0.0481
*Age*	−0.0367	0.0040^**^	*Control_Work*	−0.2152	0.0404^**^
*SBP*	−0.0150	0.0031^**^	*Ability_Usage*	−0.1039	0.0434^*^
*DBP*	0.0154	0.0044^**^	*W_Suitability*	−0.1056	0.0440^*^
*GOT*	−0.00392	0.00541	*Reward*	0.0788	0.0426
*GPT*	0.00367	0.00273	*Irritation*	0.0922	0.0359^*^
*Triglyceride*	0.0005	0.0004	*Fatigue*	−0.1605	0.0468^**^
*HDL*	−0.0049	0.0028	*Depression*	−0.0193	0.0430
*HbA1c*	0.1633	0.0592^**^	*P_Compaint*	−0.2130	0.0390^**^
*Anamnesis*	0.3078	0.0732^**^	*C_Support*	0.0702	0.0366
*M_BP*	0.3130	0.1292^*^	*F_Support*	0.1242	0.0276^**^
*M_Glucose*	−0.5824	0.2455^*^	*Satisfaction*	−0.0894	0.0497
*CBD*	−0.9947	0.5973	*Y22*	0.3046	0.0645^**^
*Heart_D*	1.0592	0.1649^**^	*Q1*	0.3458	0.0964^**^
*Kidney_D*	0.9272	0.4504^*^	*Q3*	0.3147	0.0956^**^
*Anemia*	0.0666	0.0739	*Q4*	0.6024	0.0916^**^
*Smoke*	0.5107	0.0725^**^	*Site2*	0.4763	0.1190^**^
*Weight_20*	0.3663	0.0757^**^	*Site3*	0.1312	0.0770
*Exercise*	−0.0721	0.1150	*Site4*	0.1526	0.1038
*Chew_Food*	0.0581	0.0868			
*Eat_fast*	−0.0640	0.0518	No. of observations		
*Alcohol_Freq*	0.4083	0.0725^**^	0: 144266, 1: 1148, total 15,574		
*Alcohol_Amount*	−0.3393	0.0558^**^	Log likelihood −3720.59		
*Sleep*	−0.1155	0.0705	McFadden *R*^2^ 0.092122		
*H_Guidance*	−0.1627	0.0764^*^	Likelihood ratio statistic 755.05		

SE: standard error, **: significant at the 1% level; *: significant at the 5% level.

**Figure 4:**
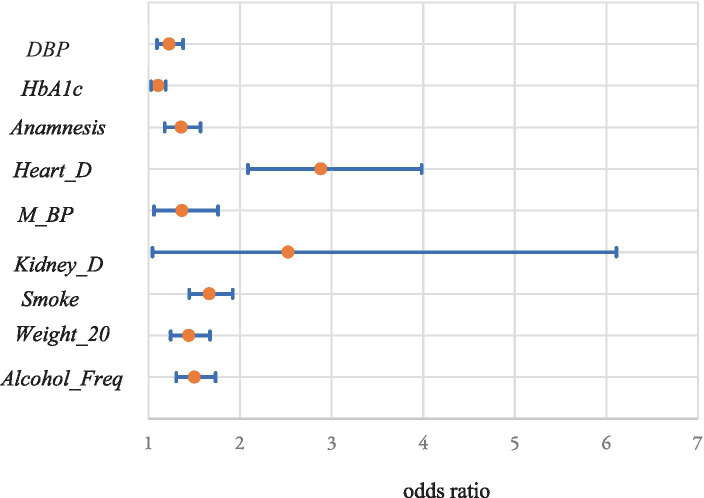
Odds ratios and 95% confidence intervals of significant variables of positive estimates obtained from medical checkups.

**Figure 5:**
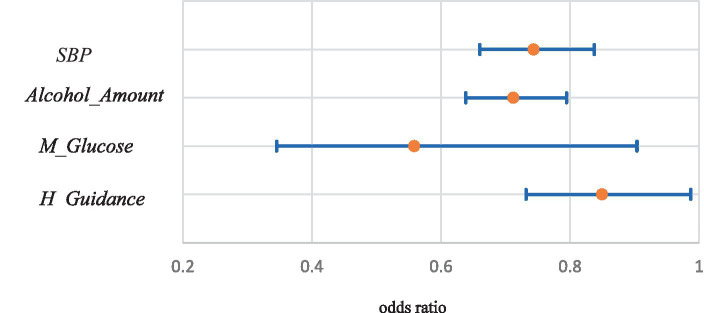
Odds ratios and 95% confidence intervals of significant variables of negative estimates obtained from medical checkups.

Among variables obtained from medical checkups, the estimates for *DBP* (OR:1.23; CI: 1.09–1.38)*, HbA1c* (1.11; 1.03–1.19)*, Anamnesis* (1.36; 1.18–1.57)*, Heart_D* (2.88; 2.09–3.98)*, Smoke* (1.67; 1.45–1.92)*, Weight_20* (1.44; 1.24–1.67), and *Alcohol_Freq* (1.50; 1.30–1.73) were positive and significant at the 1% level. The ORs and 95% CIs were given in parentheses. The ORs were calculated from one standard deviation increments of the variables for continuous variables and one point increments for discrete variables such as dummy variables. The estimates for *M_BP* (1.37; 1.06–1.76) and *Kidney_D* (2.53; 1.05–6.11) were positive and significant at the 5% level. In contrast, the estimates for *SBP* (0.74; 0.66–0.84) and *Alcohol_Amount* (0.71; 0.64–0.79) were negative and significant at the 1% level. The estimates for *M_Glucose* (0.56; 0.35–0.90) and *H_Guidance* (0.85, 0.73–0.99) were positive and significant at the 5% level. Figures 4, 5 show the ORs and 95% CIs for significant variables of positive and negative estimates obtained from medical checkups, respectively.

**Figure 6:**
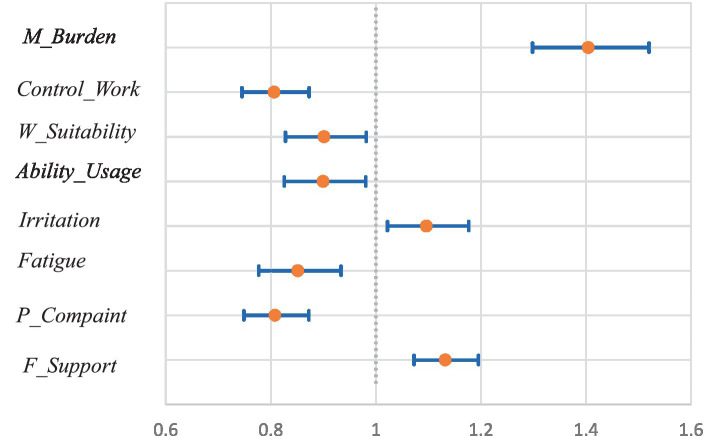
Odds ratios and 95% confidence intervals of significant variables obtained from the BJSQ.

Concerning variables obtained from the BJSQ, the estimates for *M_Burden* (1.40; 1.30–1.52) and *F_Support* (1.13; 1.07–1.20) were positive and significant at 1%, and that for *Irritation* (1.10; 1.02–1.18) was positive and significant at the 5% level. The estimates for *Control_Work* (0.81; 0.74–0.87) and *P_Complaint* (0.81; 0.75–0.87) were negative and significant at the 1% level, while those for *Ability_Usage* (0.901; 0.828–0.981) and *W_Suitability* (0.900; 0.825–0.981) were negative and significant at the 5% level. Figure 6 shows the ORs and 95% CIs for these variables.

**Figure 7:**
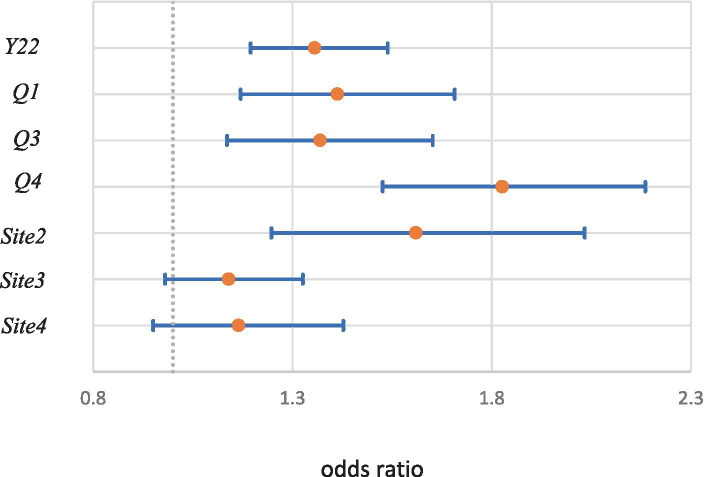
Odds ratios and 95% confidence intervals of year, seasonal, and site dummies.

Year and all quarter dummies were positive and significant at the 1% level. The ORs and 95% CIs were (1.36; 1.13–1.54), (1.41;1.17–1.71), (1.14; 1.37–1.65), and (1.83,1.53–2.19) for *Y22, Q1, Q3,* and *Q4*, respectively. For the site dummies, the estimate for *Site2* (1.61, 1.61–2.03) was positive and significant at the 1% level. Figure 7 shows the ORs and 95% CIs for these variables.

Concerning multicollinearity among covariates, the variance inflation factors (VIFs) were not large except for *SBP* (3.29), *DBP* (3.24), *GOT* (5.68) and *GPT* (6.22). The VIFs for these variables are in parentheses. Except for these variables, the largest VIF was 2.78 and not large; thus the multicollinearity problem is not particularly serious.

The correction coefficient of BP variables is relatively high (0.814). As the standard deviations (SDs) of *SBP* and *DBP* are different, we standardize them and define *S_SBP*=*SBP/s1* and *S_DBP*=*DBP/s2,* where *s1* (=19.70) and *s2* (=13.41) are the SDs of *SBP* and *DBP*. We consider the level and difference of BP as *BP_L* = (*S_SBP* + *S_DBP*)/2 and *BP_D* = *S_SBP-S_DBP*. These correspond to the first and second principal components of the standardized BP levels. The method makes the estimators for concerning variables most efficient (54) in the two variable cases. We then estimated the logit model again and obtained the results shown in Table 4.

**Table 4:** Estimation results of *BP_L* and *BP_D.*

Variable	Coefficient	Standard error	*p*-value
*BP_L*	−0.0903	0.0406	0.0261
*BP_D*	−0.2510	0.0563	0.0000

Note that these changes are simple linear transformations, and the estimation results of the other variables and the model fitness remain the same as those of Table 3. The estimate for *BP_L* was negative and not significant at the 1% level; however, the estimate for *BP_D* was negative and highly significant (*p*-value is 0.0000).

The correlation coefficient between *GPT* and *GOT* is 0.901. When we considered the level and difference between these variables, the *p*-value were 0.139 and 0.287 for the level and difference variables, respectively, and the results were not significant.

## 4 Discussion

### 4.1 Basic characteristics

Age is a significant factor affecting long-term absenteeism. However, the paid leave days given to employees depend on the number of working years at the company, and the days become longer as the working years increase [Article 39 of the Labor Standards Act (55)]. Evidently, the working experience of younger employees tends to be shorter than that of older employees. Unfortunately, working years were not available for the dataset. Therefore, additional studies concerning to age and working years are required.

Gender is an important factor. The OR for females compared to males is 2.26 [In this case, because the probability of long-term absenteeism is relatively low, the OR approximates the probability ratio (56)]. This means that the long-term absenteeism probability of females is approximately twice that of males. The corporation depends heavily on female employees. Almost three-fourths of employees are female, and their absence directly affects corporate performance. Labor and health policies aimed at female employees are necessary.

### 4.2 Variables obtained from the medical checkups

Concerning blood pressure (BP), the relationships of *SBP* and *DBP* to absenteeism are opposite. The estimate for *SBP* is negative and that for *DBP* is positive. This means that higher SBP may reduce long-term absenteeism, whereas higher DBP may increase it. The results of Table 4 suggest that the (standardized) difference between SBP and DBP does matter, but the BP level may be less important. There have been many studies on the relationship between BP and diseases (especially cardiovascular diseases) (50). Therefore, it might be necessary to reevaluate the relationship between BP and disease from this viewpoint.

*HbA1c* is positively related to absenteeism. *Anamnesis* and histories of heart disease and kidney disease may increase the probability of long-term absenteeism. Compared to those without them, the ORs are 1.36, 2.88 and 2.53 for those with anamnesis, heart disease, and kidney disease, respectively. Therefore, special healthcare by the corporation should be necessary for such employees.

Smoking habits and large weight increases can also affect absenteeism. The ORs are 1.67 and 1.44 compared to those without them. These results are consistent with those of previous studies (20, 57–59). Health guidelines may reduce absenteeism as expected. These factors are modifiable through, and it may be worthwhile for the corporation to help improve these factors. The results for alcohol consumption are mixed. If an employee drinks more frequently, it may increase absenteeism; however, if an employee drinks more alcohol at once, the probability of absenteeism may decline. We cannot determine the reasons for this finding, and further studies regarding alcohol consumption are necessary. Antihypertensive medications would increase absenteeism, but antihyperglycemic medications would decrease.

### 4.3 Variables obtained from the BJSQ

The BJSQ primarily represents employees’ mental elements. Among them, the burden concerning the quantity of work (*M_Burden*) is positive and highly significant (*t*-value = 8.45, *p*-value = 0.000). It is not a good sign, and it is reasonable to consider that employees might be overworking because they cannot take off days due to too much work. In the worst case, overwork results in employee suicide (60). Employers and managers at operational sites should pay attention to avoiding employee overwork.

*W_Control, Ability_Usage, W_Suitability* are significant at the 1% or 5% levels. These variables represent motivation, suitability, and willingness to work. Because the meanings of these variables are similar, we consider the case in which the values of all variables increase by one. ${\hat{\beta }}_{29},{\hat{\beta }}_{30}\;\mathrm{and}\;{\hat{\beta }}_{31}$ are the estimators of these variables. Since $V\left(\sum {\hat{\beta }}_{i}\right)=\sum V\left({\hat{\beta }}_{i}\right)+2\underset{i<j}{\sum }cov\left({\hat{\beta }}_{i},{\hat{\beta }}_{j}\right)$ and $cov\left({\hat{\beta }}_{i},{\hat{\beta }}_{j}\right)$ = − 0.00021, −0.00034 and − 0.00021 for (*i* = 29, *j* = 30), (*i* = 29, *j* = 31) and (*i* = 30, *j* = 31), $V\left({\hat{\beta }}_{29}+{\hat{\beta }}_{31}+{\hat{\beta }}_{32}\right)$ =0.00394. Therefore, the OR is 0.65 with 95% CI of 0.58–0.74 (the OR and CI are calculated by comparing $\sum x_{i}$ and $\sum \left(x_{i}+1\right)$ ). This finding suggests that employers and managers can reduce long-term absenteeism by one-third through suitable work arrangements that would motivate employees.

*Irritation* is significant at the 5% level. However, the sign is positive. Further studies are necessary to address employee irritation. *Fatigue* and physical complaints (*P_Complaint*) are significant at the 1% level. Compared to the one-point improvement case, the ORs become 0.85 and 0.81, suggesting that long-term absenteeism could be reduced by about 15% and 20% through one-point improvement in these factors. The estimate for support from family and friends (*F_Support*) is positive and significant at the 1% level. It is reasonable to assume that family and friends advise employees to take off days more often when conditions are poor. Surprisingly, the estimate for work and family life satisfaction is not significant at even the 5% level. Unlike the results of Bryan et al. (44), these results may imply that physical factors are more important than mental factors. Li and Wang (61) evaluated the work-family initiatives including reduced work hours, flexible schedule and telework. They found that the mental health benefits of flexible schedule and telework initiatives were larger than reduced hours initiatives. It may be worthwhile for the government and employers to promote flexible work time systems to reduce employees’ physical burdens and complaints.

### 4.4 Year, quarter, and site dummies

The estimate of the dummy variable for 2022 (*Y22*) is highly significant, suggesting a large difference in 2021 and 2022. The average daily number of new coronavirus patients (COVID-19) patients in Japan was 4,010 in February–December 2021, and 75,175 in January–December 2022 (62). This huge difference might have affected absenteeism; however, further investigation is necessary to evaluate the relationship of COVID-19 to absenteeism.

All estimates for quarter dummies are significant. The seasonal factors are important, especially in the fourth quarter (*Q4*, October–December). The operational sites are located in the northeastern region of Japan. The first snowfall occurs in mid-November; moreover, the daytime becomes shorter, and the weather becomes colder daily in the fourth quarter, which might affect the behaviors of employees. The estimate for *Site2* is positive and significant. The percentages of observations answered the BJSQ are 93.5, 43.6, 94.2, and 93.4% at Sites 1, 2, 3, and 4. The value of Site 2 is the smallest, which might affect employees’ labor and health management.

## 5 Conclusion

This study analyzed the physical and mental health factors of employees that may be related to absenteeism. The dataset included the results of annual medical checkups, BJSQ, and work records at four operational sites in a large corporation. The sample period was from February 2021 to January 2022. Because there were too many potential covariates, health-related covariates were selected using the stepwise procedure. Subsequently, 15,574 observations from 2,319 employees were used in a logistic regression (logit) model.

The long-term absenteeism probability for females was much higher than that for males. The corporation depends heavily on female employees. Labor and health policies aimed at female employees are necessary for the corporation. The opposite relations were observed for SBP and DBP. These results suggest that the (standardized) difference between SBP and DBP was more important than the BP level. HbA1c, anamnesis and histories of heart disease and kidney disease were positively related to the probability of long-term absenteeism. Smoking habits and large weight increments were positively associated with absenteeism. Health guidelines might reduce the absenteeism. It may be worthwhile for the corporation to help improve them. The results for alcohol consumption were mixed. Antihypertensive medications would increase absenteeism but antihyperglycemic medications would decrease.

Among the BJSQ vaeiables, the quantity of work was positive and highly significant, and employers and managers should pay attention to avoiding overworking employees. Improving workers’ motivation through suitable work arrangements could reduce long-term absenteeism by one-third. Fatigue and physical complaints were also important, and long-term absenteeism cloud be reduced by improving physical conditions. The estimate of support from family and friends was positive and significant. However, the estimate of work and family life satisfaction was not significant even at the 5% level.

The estimate of the dummy variable for 2022 was highly significant. Therefore, COVID-19 might have affected absenteeism. Seasonal factors were important, particularly in the fourth quarter. The estimate for *Site2* was positive and significant, and it may be necessary to revise the labor and health management policies at the site. Among the major countries, the Japan is the only country performing annual mandatory health checkups and job stress checks for most employees regardless of their health conditions (63). It is extremely costly to do such a survey in other countries. The results of the paper would help when the similar types of studies or policy analyses are done in other countries.

The results of this study are based on operational sites of one corporation and the dataset was observatory. The employees were mainly operators working inside the buildings, and most of them are healthy people. Therefore, the sample selection biases might exist, and results may differ for different working conditions, job types, or companies. Hence, the results of this study cannot be generalized. However, annual medical checkups and the BJSQ for employees are mandatory for most companies, and the framework of this study is applicable to most companies in Japan. The influence of presenteeism is not evaluated. The implementations to improve the employees’ health conditions are also important. These are the limitations of the study and should be investigated in future studies.

## Data availability statement

The data were provided to the author under the official agreement of Hitotsubashi University and the corporation. The data for this study are not publicly available to protect personal information. A new agreement with the corporation is necessary for future research. Researchers may contact the corresponding author with the dials of agreement.

## Ethics statement

The studies involving humans were approved by Institutional Review Board of Hitotsubashi University (2022C003). The studies were conducted in accordance with the local legislation and institutional requirements. Written informed consent for participation in this study was provided by the participants’ legal guardians/next of kin.

## Author contributions

KN: Conceptualization, Data curation, Formal analysis, Investigation, Methodology, Project administration, Resources, Software, Supervision, Visualization, Writing – original draft, Writing – review & editing.
