# Supplementary material for: Evaluation of physical and mental health conditions related to employees’ absenteeism
Source: Front Public Health. 2024 Jan 11;11:1326334. doi: 10.3389/fpubh.2023.1326334 (PMC10808730; doi:10.3389/fpubh.2023.1326334)
Supplement: Supplementary file 1 [file Table_1.pdf]

Appendix A. Variables not used in Equation (5)

The following 22 variables were not included as covariates in Equation (5) based on the variable selection procedure.

Variables obtained from medical checkups:

Height, weight, body mass index, number of red blood cells, hemoglobin, γ-glutamyl transferase, low-density lipoprotein cholesterol, blood sugar, subjective symptoms, cholesterol medications, performing physical activity (walking or equivalent) for one hour or more, walking speed, late supper, snaking, no breakfast, and lifestyle improvement.

Variable obtained from the BJSQ:

Mental burden of work (quality), stress due to personal relationships at work, stress due to the work environment, vitality, anxiety, and support from supervisors.

Appendix B. Abbreviations

Table A.1 gives the list abbreviations used in this paper.

Table A.1 List of abbreviations

Symbol	Meaning	Symbol	Meaning
Female	female	*Alcohol_Freq*	frequency of alcohol intake
Age	age	*Alcohol_Amount*	amount of alcohol intake
SBP	systolic blood pressure	*Sleep*	sleeping well
DBP	diastolic blood pressure	*H_Guidance*	will take health guidance
GOT	glutamic-oxaloacetic transaminase	*M_Burden*	amount of work felt
GPT	glutamic-pyruvic transaminase	*S_P_Burden*	subjective physical burden
Triglyceride	serum triglyceride level	*Control_Work*	control level of work
HDL	high-density lipoprotein cholesterol	*Ability_Usage*	utilization of knowledge and skills at work
HbA1c	hemoglobin A1c	*W_Suitability*	suitability of the work
Anamnesis	having anamnesis	*Reward*	rewarding work
M_BP	taking antihypertensive medications	*Irritation*	irritation
M_Glucose	taking antihyperglycemic medications	*Fatigue*	fatigue
CBD	cerebrovascular disease history	*Depression*	depression
Heart_D	heart disease history	*P_Compaint*	physical complaints
Kidney_D	kidney disease history	*C_Support*	support from co-workers
Anemia	having anemia	*F_Support:*	support from family and friends
Smoke	smoking	*Satisfaction*	work and family life satisfaction
Weight_20	weight gain from age 20	*Y22*	year dummy for 2022
Exercise	exercising	*Q1,Q3,Q4*	quarter dummies
Chew_Food	can chew food items	*Site2,Site3,Sit4*	site dummies
Eat_fast	eating speed		
